# Quantitative Evaluation of Burn Injuries Based on Electrical Impedance Spectroscopy of Blood with a Seven-Parameter Equivalent Circuit

**DOI:** 10.3390/s21041496

**Published:** 2021-02-21

**Authors:** Huilu Bao, Jianping Li, Jianming Wen, Li Cheng, Yili Hu, Yu Zhang, Nen Wan, Masahiro Takei

**Affiliations:** 1 The Institute of Precision Machinery and Smart Structure, College of Engineering, Zhejiang Normal University, Jinhua 321004, China; baohuilu@zjnu.edu.cn (H.B.); wjming@zjnu.cn (J.W.); chenglizjnu@foxmail.com (L.C.); huyili@zjnu.edu.cn (Y.H.); zjnuzy@zjnu.cn (Y.Z.); wannen@zjnu.cn (N.W.); 2 Graduate School of Mechanical Engineering, Division of Artificial System Science, Chiba University, 1-33 Yayoi, Inage, Chiba 263-8522, Japan; masa@chiba-u.jp

**Keywords:** burn injury, electrical impedance spectroscopy, blood, red blood cell

## Abstract

A quantitative and rapid burn injury detection method has been proposed based on the electrical impedance spectroscopy (EIS) of blood with a seven-parameter equivalent circuit. The degree of burn injury is estimated from the electrical impedance characteristics of blood with different volume proportions of red blood cells (RBCs) and heated red blood cells (HRBCs). A quantitative relationship between the volume portion *H*_HCT_ of HRBCs and the electrical impedance characteristics of blood has been demonstrated. A seven -parameter equivalent circuit is employed to quantify the relationship from the perspective of electricity. Additionally, the traditional Hanai equation has been modified to verify the experimental results. Results show that the imaginary part of impedance *Z*_Imt_ under the characteristic frequency (*f*_c_) has a linear relationship with *H*_HCT_ which could be described by *Z*_Imt_ = −2.56*H*_HCT_ − 2.01 with a correlation coefficient of 0.96. Moreover, the relationship between the plasma resistance *R*_p_ and *H*_HCT_ is obtained as *R*_p_ = −7.2*H*_HCT_ + 3.91 with a correlation coefficient of 0.96 from the seven -parameter equivalent circuit. This study shows the feasibility of EIS in the quantitative detection of burn injury by the quantitative parameters *Z*_Imt_ and *R*_p_, which might be meaningful for the follow-up clinical treatment for burn injury.

## 1. Introduction

It is reported that about 11 million people suffer from burn injuries every year all around the world [1,2,3]. The diagnosis of burn injury degrees which could provide an important reference for clinical treatment has always been an important part of burn injury studies. The quantitative measurement of burn injury is still a hot topic in the fields of research and clinical treatment.

Nowadays, some methods have been developed by researchers to measure the degrees of burn injury. The observation method is one of the most common utilized methods with an advantage of low cost [4]. However, since it is measured by the eyes of doctors, the result is greatly influenced by personal experience which may lead to a low accuracy [5]. The pathological biopsy method (PB) is another method which means the removal of diseased tissue, and its accuracy is generally high. Nevertheless, it brings harm to patients’ body, and it also takes a long time to determine the degrees of burn injury [6,7]. Sometimes, other methods, like fluorescence detection (FD), infrared thermal imaging (ITI), ultrasonic imaging (UI) and computed tomography (CT) are also applied for the measurement of burn injury. However, all these methods have their own advantages and disadvantages [4,5,8,9]. New methods with high efficiency, high speed, low cost and less body harm are still necessary to be investigated to save the life and time of patients with burn injury.

In recent years, the EIS method has gained the attention of researchers for its rapid speed, non-invasive and low cost measurement characteristics [10,11,12,13,14]. It has been confirmed that EIS is sensitive to the microscopic compositions of cells and proteins (such as concentration, cell size, survival state) by measuring the variation of electrical impedance characteristics [15,16]. EIS has been utilized in many aspects of medical monitoring, such as the thrombus detection [17]; myoglobin detection [18]; circulating tumor cell detection [19]; status of tissue or cell detection [20,21]. It is reported that the membrane of heated red blood cells (HRBCs) is destroyed in patients with burn injury, and different degrees of burn injury lead to different proportions of red blood cells (RBCs) and HRBCs which causes the variation of blood’s electrical impedance characteristics [22]. Wu et al. [23] pointed out that the hematocrit (*HCT*) increased as the patients gradually recovered. Therefore, it is feasible to exploit EIS method to determine the degrees of burn injury by measuring the impedance of blood with different *HCT* and the hematocrit of HRBCs (*H*_HCT_). Nevertheless, how to quantitatively evaluate the degrees of burn injury by EIS method, and which parameters are suitable for the quantitative measurement are still unknown to the real application.

In this study, to explore a new method for burn injury detection and investigate the effective parameters, a quantitative and rapid measurement has been proposed based on EIS method by detecting the proportion of HRBCs. The content of this paper is as the following: in Section 2, an experiment system is built up to detect the electrical impedance characteristics of blood with different *HCT* and *H*_HCT_. In Section 3, a seven-parameter equivalent circuit is employed to quantify the relationship from the perspective of electricity. Additionally, the conventional Hanai equation has been modified to quantify the relationship. This study might be meaningful to a more efficient, economical and rapid burn injury detection method.

## 2. Experiment

### 2.1. Experimental Setup

**Figure 1:**
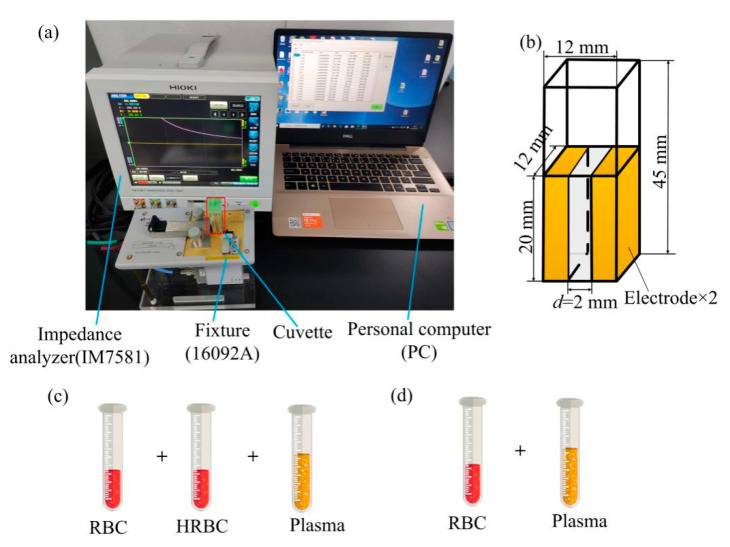
(**a**) Experimental setup; (**b**) structural dimension of the cuvette; (**c**) sample preparation of blood with different burn injury degrees (plasma, RBCs and HRBCs); (**d**) sample preparation of blood with different concentrations of RBCs.

The experimental setup for the burn injury detection by EIS method is shown in Figure 1a. It mainly consists of a cuvette, an impedance analyzer (IM7581, Hioki E.E. Corporation, Nagano-ken, Japan) with a fixture (16092A, Agilent Technologies Inc., Palo Alto, CA, USA), and a personal computer (PC). In the experimental setup, blood is put inside the cuvette with two electrodes which are fabricated by copper. The structural dimension parameters of the cuvette are 12 mm × 12 mm × 45 mm, and the size of the electrodes in the cuvette is 10 mm × 20 mm with a distance of 2 mm, as is shown in Figure 1b. During the experiment, the impedance analyzer is utilized to measure the electrical impedance parameters of blood with the cuvette fixed in the fixture. The PC is used to process the data from the impedance analyzer.

### 2.2. Sample Preparation

Swine blood is utilized in this study instead of human blood because of the ethical and regulatory considerations. The swine blood is taken from a slaughterhouse within 12 h. A trisodium citrate solution with a concentration of 3.28% is injected into the swine blood with a volume ration of 1:9 to prevent blood clotting. Before the experiment, swine blood is put into the thermostatic water bath (HH-4, Jintan Chengdong Xinrui Instrument Factory, Changzhou, China) at 55 ℃ for 1 h to get the heated swine blood, which has been confirmed to be effective to mimic the burn injury for blood [24]. Then, the heated swine blood is put into the centrifuge to get the HRBCs, and normal swine blood is put into the centrifuge to get RBCs and plasma after centrifuging at 2000× *g* rpm for 30 min under room temperature. Two experiments are designed to measure the difference between the electrical impedance characteristics of RBCs and HRBCs. As illustrated in Figure 1c, experiment is to mimic the blood with different burn injury degrees by mixing plasma with RBCs, HRBCs of different proportions. In the second experiment, plasma is mixed with RBCs to mimic the normal blood, as is shown in Figure 1d.

### 2.3. Experimental Results

**Figure 2:**
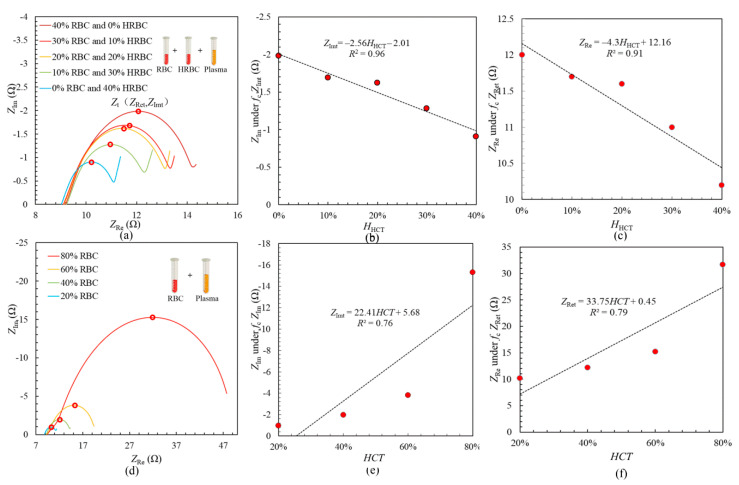
(**a**) The Nyquist plots of the blood with different *HCT* and *H*_HCT_; (**b**) Relationship between *H*_HCT_ and *Z*_Imt_; (**c**) Relationship between *H*_HCT_ and *Z*_Ret_; (**d**) The Nyquist plots of the blood with different *HCT*; (**e**) Relationship between *HCT* and *Z*_Imt_; (**f**) Relationship between *HCT* and *Z*_Ret_.

Figure 2a illustrates the experimental results about the impedance characteristics of blood with different proportions of HRBCs and RBCs measured by the system in Figure 1a. The experiments were carried out under the conditions of room temperature *t* = 25 °C. By inserting different volumes of RBCs and HRBCs into plasma, the obtained *HCT* is 0%, 10%, 20%, 30%, 40%, and *H*_HCT_ is 40%, 30%, 20%, 10%, 0% which ensures the the value of ‘*HCT* + *H*_HCT_’ is always 40%. It has been reported that different degrees of burn injury bring different *HCT* and *H*_HCT_ in blood [23]. Hence, the blood for the first experiment is mixed by different *HCT* and *H*_HCT_ to mimic blood with different injury degrees. The experiments were taken under the sweeping frequency from *f* = 100 kHz to *f* = 300 MHz and the applied current of *I* = 10 mA. It is to note that because of the effect of inductance, some experimental date whose imaginary part of impedance is larger than 0 is going to be not shown in the figures. As shown in Figure 2a, the Nyquist plots of the blood with different *HCT* and *H*_HCT_ all follow the classical shape of a semicircle: as frequency *f* goes up, the value of impedance increases at low frequencies and then it decreases after the characteristic frequency (peak point *Z*_t,_ the impedance under characteristic frequency *f*_c_). To clearly clarify the electrical impedance characteristic variation, the peak point *Z*_t_ (*Z*_Ret_, *Z*_Imt_) of each Nyquist plot is investigated. Here, *Z*_Ret_ is the real part of impedance under the characteristic frequency *f*_c_, *Z*_Imt_ is the imaginary part of impedance under the characteristic frequency *f*_c_. The radius of the Nyquist plot has a negative correlation with *H*_HCT_, and the value of *Z*_Imt_ reduces from 1.98 Ω to 0.91 Ω under the condition that *H*_HCT_ is from 0% to 40%. Moreover, as is seen in Figure 2b, the linear relationship between *Z*_Imt_ and *H*_HCT_ is shown as: *Z*_Imt_ = −2.56 *H*_HCT_ + 2.01 with a great linearity of *R*^2^ = 0.96. Figure 2c shows the linear relationship between *Z*_Ret_ and *H*_HCT_ which is obtained as: *Z*_Ret_ = −4.3 *H*_HCT_ +12.16 with a linearity of *R*^2^ = 0.91. Compared with linearity of two relationships, *Z*_Imt_ is more sensitive to H*_HCT_*. According to previous studies [23,25], *H*_HCT_ is related to the degrees of burn injury. It means that *Z*_Imt_ might be an effective parameter to detect the degree of burn injury.

In order to further investigate the parameter *Z*_Imt_ and the influence of *HCT*, the second experiment is conducted with the obtained *HCT* changing from *HCT* = 20% to *HCT* =80%. As shown in Figure 2c, the Nyquist plots of blood with different *HCT* also follow the shape of a semicircle. The radius of Nyquist plot increases under the condition that *HCT* enhances. Additionally, the value of *Z*_Imt_ climbs from 0.98 Ω to 15.3 Ω in the case that *H*_HCT_ jumps from 20% to 80%. The relationship between *Z*_Imt_ and *HCT* is shown as: *Z*_Imt_ = 22.41 *HCT* +5.68 with a low linearity of *R*^2^ = 0.76, which means *Z*_Imt_ is not so sensitive to *HCT*. Similarly, in Figure 2f, relationship between *Z*_Ret_ and *HCT* is shown as: *Z*_Ret_ = 33.75 *HCT* +0.45 with a low linearity of *R*^2^ = 0.79, which is also not sensitive to *HCT*. As a result, *Z*_Imt_ is confirmed to be effective to measure *H*_HCT_, which means that it could be treated as a quantitative parameter to measure the degrees of burn injury.

## 3. Discussion

### 3.1. Equivalent Circuit

**Figure 3:**
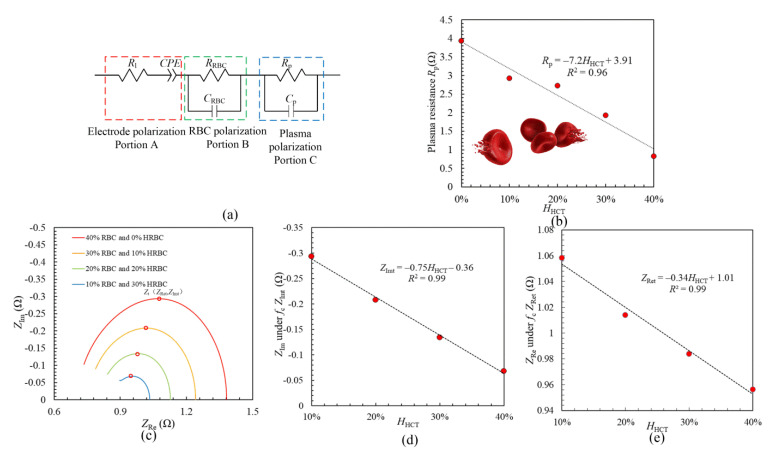
(**a**) Seven-parameter equivalent circuit of blood; (**b**) Fitting results: HRBC and relationship between *H*_HCT_ and *R*_p_; (**c**) The Nyquist plots of blood with different *HCT* and *H*_HCT_; (**d**) Relationship between *H*_HCT_ and *Z*_Imt_; (**e**) Relationship between *H*_HCT_ and *Z*_Ret_.

In order to obtain the information of the electrical impedance properties of blood with different degrees of burn injury, the electrical impedance system is described by the seven -parameter equivalent circuit in Figure 3a. The blood system is divided into three parts: electrode polarization portion A, RBC polarization portion B and plasma portion C. *R*_1_ is the electrolyte resistance; *C*_RBC_ is the RBC capacity, *R*_RBC_ is the resistance of RBC; *R*_p_ is the resistance of plasma and *C*_p_ is the capacitance of plasma. The impedance of the CPE is [26,27]:

(1) $$Z_{\mathrm{CPE}}=\frac{\cos(\frac{\pi }{2})p}{{\left(2\pi f\right)}^{p}T}-j\frac{\sin(\frac{\pi }{2})p}{{\left(2\pi f\right)}^{p}T}$$

where CPE is a ideal capacitor if *p* is close to 1 or a resistor if *p* is close to 0; *t* is the CPE constant, *j* is the imaginary unit, 2*πf* is angular frequency.

By taking the seven-parameter equivalent circuit, the fitting results in Figur 3b show that there is a great linear relationship between *H*_HCT_ and plasma resistance *R*_p_. Table 1 shows the fitting parameters. The value of *R*_p_ goes up from *R*_p_ = 3.93 Ω to *R*_p_ = 0.83 Ω in the case that H_HCT_ increases from *H*_HCT_ = 0% to *H*_HCT_ = 40%. Moreover, the relationship between *R*_p_ and *H*_HCT_ is obtained as: *R*_p_ = −7.2*H*_HCT_ +3.91 with a linearity of *R*^2^ = 0.96. This is thought to be caused by the fact that burn injury leads to the rupture of HRBCs. The capacitive properties of the cell membrane decreases after the rupture of HRBCs, and the hemoglobin inside cells escapes into the plasma which enhances the resistive properties of the plasma. From the proposed seven-parameter equivalent circuit, it is found that the parameter plasma resistance R_p_ is also effective to be applied as an indicator of burn injury degrees.

### 3.2. Modified Hanai Equation

In this research, in order to quantitatively analyze the impedance variation of blood with different degrees of burn injury, traditional Hanai equation is modified for the theoretical calculation. Hanai equation is widely applied for the electrical impedance calculation in heterogeneous systems of dense colloids. The conventional Hanai equation has already been modified from the viewpoint of different proportions of RBCs and HRBCs. Moreover, from the previous experimental results in Section 2, the blood is simplified as the solution of RBCs, HRBCs and plasma. The RBCs and HRBCs have been simplified as the 2-dimensional elliptical cells.

Here, modified Hanai equation with the elliptical model including all these influencing factors is calculated as follows:

(2) $$\left(\frac{\varepsilon_{\mathrm{plasma}}^{*T}-\varepsilon_{\mathrm{RBC}}^{*}}{\varepsilon_{\mathrm{plasma}}^{*}-\varepsilon_{\mathrm{RBC}}^{*}}\right){\left(\frac{\varepsilon_{\mathrm{plasma}}^{*T}}{\varepsilon_{\mathrm{plasma}}^{*}}\right)}^{-C_{1}}{\left(\frac{\varepsilon_{\mathrm{plasma}}^{*T}+A^{\prime }\varepsilon_{\mathrm{RBC}}^{*}}{\varepsilon_{\mathrm{plasma}}^{*}+A^{\prime }\varepsilon_{\mathrm{RBC}}^{*}}\right)}^{-C_{2}}{\left(\frac{\varepsilon_{\mathrm{plasma}}^{*T}+B^{\prime }\varepsilon_{\mathrm{RBC}}^{*}}{\varepsilon_{\mathrm{plasma}}^{*}+A^{\prime }\varepsilon_{\mathrm{RBC}}^{*}}\right)}^{-C_{3}}=1-HCT$$

(3) $$\left(\frac{\varepsilon_{\mathrm{blood}}^{*}-\varepsilon_{\mathrm{HRBC}}^{*}}{\varepsilon_{\mathrm{plasma}}^{*T}-\varepsilon_{\mathrm{HRBC}}^{*}}\right){\left(\frac{\varepsilon_{\mathrm{blood}}^{*}}{\varepsilon_{\mathrm{plasma}}^{*T}}\right)}^{-C_{1}}{\left(\frac{\varepsilon_{\mathrm{blood}}^{*}+A^{\prime }\varepsilon_{\mathrm{HRBC}}^{*}}{\varepsilon_{\mathrm{plasma}}^{*T}+A^{\prime }\varepsilon_{\mathrm{HRBC}}^{*}}\right)}^{-C_{2}}{\left(\frac{\varepsilon_{\mathrm{blood}}^{*}+B^{\prime }\varepsilon_{\mathrm{HRBC}}^{*}}{\varepsilon_{\mathrm{plasma}}^{*T}+B^{\prime }\varepsilon_{\mathrm{HRBC}}^{*}}\right)}^{-C_{3}}=1-H_{\mathrm{HCT}}$$

where $\varepsilon_{\mathrm{blood}}^{*}$ is the complex permittivity of blood, $\varepsilon_{\mathrm{plasma}}^{*}$ is the complex permittivity of cell-free plasma and $\varepsilon_{T}^{*}$ plasma is the complex permittivity of plasma with RBCs; $\varepsilon_{\mathrm{RBC}}^{*}$ is the complex permittivity of RBC and $\varepsilon_{\mathrm{HRBC}}^{*}$ is the complex permittivity of HRBC; *A*^′^ and *B*^′^ are the orientation factors influenced by RBCs orientation. *C*^1^, *C*^2^, *C*^3^ are the deformation factors influenced by electrical field. $\varepsilon_{m}^{*}$ is the relative permittivity of membrane. *σ*_plasma_ and *σ*_m_ are the conductivity of plasma and membrane, respectively. The other values based on the elliptical cell model have been described as:

(4) $$\varepsilon_{\mathrm{plasma}}^{*}=\varepsilon_{\mathrm{plasma}}-j\frac{\sigma_{\mathrm{plasma}}}{\omega \varepsilon_{0}}$$

(5) $$\varepsilon_{m}^{*}=\varepsilon_{m}-j\frac{\sigma_{m}}{\omega \varepsilon_{0}}$$

Based on the modified Hanai equation [17], the impedance Z^*^ of blood is achieved according to Equation (6):

(6) $$Z^{*}={\left(\frac{1}{d}j2\pi fA\varepsilon_{\mathrm{blood}}^{*}\right)}^{-1}$$

where *A* is the area of the electrode of the container; *d* is the distance between the two electrodes of container, which is shown in Figure 1b.

### 3.3. Calculation Results

Figure 3c illustrates the calculation results of the impedance characteristics with different *HCT* and *H*_HCT_ based on the modified Hanai equation. The Nyquist plots of blood with different *HCT* and *H*_HCT_ all have semicircles, and the radius of the Nyquist plot reduces as *H*_HCT_ enhances. The value of impedance climbs at low frequencies and then it decreases after the characteristic frequency *f*_c_ (peak point *Z*_t_). To clearly clarify the electrical impedance characteristic variation, the peak point *Z*_t_ (*Z*_Ret_, *Z*_Imt_) of each Nyquist plot is also investigated. The value of *Z*_Imt_ reduces from 0.29 Ω to 0.07 Ω under the condition that *H*_HCT_ goes up from 10% to 40% in the calculation results. Figure 3d illustrates *Z*_Imt_ is linearly related to *H*_HCT_, moreover, the linear relationship between them is shown as *Z*_Imt_ = −0.75*H*_HCT_ +0.36. This linear relationship has a great linearity of *R*^2^ = 0.99. The linear relationship between *Z*_Ret_ and *H*_HCT_ is illustrated in Figure 3e as: *Z*_Ret_ = −0.34*H*_HCT_ + 1.01 with a great linearity of *R*^2^ = 0.99. According to the calculation results in Figure 3 and the experimental results in Figure 2, it is confirmed that the parameter *Z*_Imt_ has a great linear relationship with *H*_HCT_, which is effective to measure the degrees of burn injury quantitatively.

## 4. Conclusions

In this study, a quantitative and rapid burn injury detection method based on EIS has been proposed. The degrees of burn injury are estimated from the impedance values of blood with different *HCT* and *H*_HCT_. Results show that the parameter ***Z*_Im_**_t_ and *R*_p_ are effective to be applied for the quantitative detection of burn injury. The linear relationship between ***Z*_Im_**_t_ and *H*_HCT_ could be obtained as ***Z*_Im_**_t_ = −2.56*H*_HCT_ −2.01 with a high approximation of *R*^2^ = 0.96. Additionally, the linear relationship between plasma resistance *R*_p_ and *H*_HCT_ could be obtained as *R*_p_ = −7.2*H*_HCT_ +3.91 with a high approximation of *R*^2^ = 0.96. In the future, more work is still needed to make the practical application for burn injury patients.

## Figures and Tables

**Table 1:** Fitting parameters.

*H*_HCT_ (-)	*R*_1_ (Ω)	*CPE*_-T_ (F·sec^−0.28^)	*p*_-T_ (-)	*R*_RBC_ (Ω)	*C*_RBC_ (F)	*R*_P_ (Ω)	*C*_p_ (F)
40%	9.2	0.55	0.72	0.9	0.02	0.83	0.01
30%	9.2	0.55	0.72	0.8	0.01	1.93	0.02
20%	9.2	0.55	0.72	0.85	0.01	2.73	0.018
10%	9.2	0.55	0.72	0.75	0.01	2.93	0.02
0	9.2	0.55	0.72	0.5	0.005	3.93	0.01
